# Forensic assertive community treatment: an emerging best practice

**DOI:** 10.1017/S1092852925000069

**Published:** 2025-02-20

**Authors:** J. Steven Lamberti, Robert L. Weisman

**Affiliations:** 1 Chair, Research Subject Review Board for Behavioral and Social Sciences, Professor of Psychiatry, University of Rochester Medical Center, Rochester, NY, USA; 2 Director, Charles E. Steinberg Memorial Fellowship in Psychiatry and Law, Professor of Psychiatry, University of Rochester Medical Center, Rochester, NY, USA

**Keywords:** assertive community treatment, criminal justice, community mental health, forensic assertive community treatment, forensic

## Abstract

People with serious mental illness (SMI) are over-represented throughout the US criminal justice system. To address this issue, forensic assertive community treatment has recently emerged as a best-practice intervention. Also known as forensic ACT, ForACT, or most commonly as “FACT,” forensic assertive community treatment is an adaptation of the assertive community treatment (ACT) model. Unlike ACT, however, FACT is purposefully designed to prevent arrest and incarceration among people with SMI who have histories of involvement with the criminal justice system (i.e., “justice-involved” individuals). Although FACT was recognized as a best practice by the Substance Abuse and Mental Health Services Administration (SAMHSA) in 2019, FACT teams vary widely in their structure and daily operations. This lack of a standard FACT model continues to impede FACT program implementation and outcomes research. This article begins with a review of FACT origins, followed by a discussion of what we know (and do not know) about FACT operation and effectiveness. Based on the authors’ experience, the article then discusses key components of FACT and concludes with a discussion of current challenges and research recommendations for FACT model development.

## Introduction

Prior to the 1950’s, people with serious mental illness (SMI) such as schizophrenia, schizoaffective disorder, and bipolar disorder were primarily served by large state psychiatric hospitals. Between 1955 and 1976, approximately 70% of state and county psychiatric hospital inpatients were discharged into communities across America,^1^ many of which lacked the necessary services to care for them in community settings. To address the special needs of formerly institutionalized patients with SMI, the assertive community treatment (ACT) model was developed in the 1970’s in Madison, Wisconsin following the downsizing of Mendota State Hospital.^2^ Unlike traditional outpatient treatment which requires individuals to travel to clinics for care, ACT teams engage and serve people in their preferred community settings. In addition to providing outreach, several characteristics distinguish ACT teams from traditional clinic-based mental health teams. ACT teams are intensively staffed, with a service provider-to-recipient ratio of approximately 1:10. These teams typically include a psychiatrist, nurses, social workers, and a licensed chemical dependency counselor, with many teams also including peer providers, housing specialists, family specialists, and/or vocational specialists. Given the comprehensive scope of ACT services, the ACT model is sometimes referred to as “a hospital without walls”.^3^ To promote ACT model dissemination, core ACT criteria were identified and incorporated into standardized fidelity scales including the Dartmouth Assertive Community Treatment Scale (DACTS)^4^ and the Tool for Measurement of Assertive Community Treatment (TMACT).^5^ Research showed that high-fidelity ACT teams were effective at preventing psychiatric hospitalization, reducing homelessness, and improving engagement in treatment.^6^ ^-^^8^ Subsequent research studies, however, consistently showed that ACT teams were not effective at preventing arrest or incarceration.^9^ ^-^^13^

## FACT origins

Despite questions about ACT’s effectiveness in addressing criminal justice outcomes, some ACT team clinicians continued applying and adapting the ACT model to better serve justice-involved individuals who were enrolled in their ACT teams. By the mid-1990’s, published reports began to emerge from ACT teams that exclusively served clients with criminal histories,^14^ ^,^^15^ a clear departure from the original ACT model. Some of these teams began calling themselves *FACT* teams,^16^ but it remained unclear whether or how these teams differed from standard ACT teams. One early FACT team was Project Link which began in Rochester, NY, following the downsizing of the local state hospital, Rochester Psychiatric Center (RPC).^17^ As RPC’s census declined, county officials began documenting an alarming increase of people with SMI entering the county jail. To address this issue, the authors obtained county funding in 1995 to begin Project Link, a mobile treatment team that provided in-reach to incarcerated adults with SMI to engage them in care. All referrals to Project Link came from the county jail and court system, so team clinicians began working closely with judges, court staff, and probation officers to prevent their service recipients’ re-arrest. Analysis of pre-post enrollment data showed significant reductions in both incarceration and hospitalization, along with improved community functioning.^18^ Project Link received the American Psychiatric Association’s Services Achievement Gold Award in 1999 as an innovative service delivery model.^19^ These experiences prompted the authors to consider whether similar teams were operating in other states.

To address this question, the authors conducted a national survey study in collaboration with the National Association of County Behavioral Health Directors (NACBHD). Over 300 NACBHD members were surveyed to identify ACT teams that (1) served only justice-involved patients, (2) had a criminal justice agency as their primary referral source, and (3) partnered with criminal justice agencies to perform jail diversion. Team members were subsequently interviewed to ensure that their teams met both ACT fidelity criteria and FACT study criteria. A total of 16 FACT teams in nine states were identified, but significant differences were noted in their structure and daily operations. The authors concluded that FACT was an emerging model of care, and that research was needed to better define the model and test its effectiveness. The resulting paper was published in 2004 as the first formal study of FACT, coining the term “forensic assertive community treatment” in the literature ^16^ ^,^^20^. The authors published a follow-up national survey study in 2011 and noted that the number of FACT teams had nearly doubled by that time.^21^ Most recently, findings from a 2024 national ACT survey study indicate that FACT may now be present in 19 states.^22^

The number of FACT teams currently operating in the United States is unknown, but the number is likely to be significant. According to state officials, for example, 20 FACT teams are currently operating in the states of New York and Ohio alone.^23^ ^,^^24^ In addition, research reports have described the application of ACT to forensic and justice-involved populations in Canada,^25^ the Netherlands,^26^ New Zealand,^27^ and Belgium.^28^ These reports have continued to raise basic questions about the nature of FACT and its effectiveness in serving justice-involved adults with SMI.

## What do we know (and not know) about FACT operation and effectiveness?

At least four randomized controlled trials of FACT effectiveness have been conducted to date. First, in a 2-year study comparing FACT with treatment as usual in 235 individuals with SMI facing either misdemeanor or felony charges, FACT patients spent fewer days in jail but the difference was not significant.^29^ The FACT also group had significantly more bookings, likely due to a combination of sanctions and new crimes, and no differences in convictions for new crimes between FACT and usual care. Second, in a 2-year study comparing FACT with treatment as usual in 134 incarcerated adults with SMI, FACT patients had fewer bookings, fewer psychiatric hospital days, and more outpatient treatment contacts.^30^ The FACT group also had fewer jail days but the difference was not significant, and no differences in convictions were found. Increased outpatient costs were partially offset by decreased hospital and jail costs, but no significant differences were found in overall costs between FACT and usual care. Third, a 1-year NIMH-funded study compared FACT with outpatient clinic treatment plus intensive case management in a group of 70 adults with psychotic disorders who were arrested for misdemeanor crimes.^31^ Patients receiving FACT had significantly fewer days in jail, fewer convictions for new crimes, and fewer days in the hospital along with significantly increased engagement in outpatient mental health services. A subsequent return-on-investment analysis of outcome data found a $1.50 return for every $1 spent on FACT treatment.^32^ Lastly, in 2019 the authors conducted a randomized controlled trial of two FACT teams in Minneapolis and St. Paul, MN. Recruitment was halted early due to COVID-19 and riots following George Floyd’s murder, and data were analyzed on the remaining 40 study participants. Although limited by the small sample size, preliminary data analysis suggested that FACT intervention was associated with 88% fewer days in jail and prison compared to standard care which included an ACT option.^33^

Three comprehensive literature reviews have also examined FACT effectiveness. The first, a 2016 review by Marquant et al., revealed “limited yet promising evidence in support of the effectiveness of forensic ACT for forensic outcome measures”.^34^ The authors also stated that the evidence for FACT’s effectiveness in achieving non-forensic outcomes such as reduction of hospitalization “is even more limited.” A second literature review was published in 2020 by Cuddeback and colleagues.^35^ It concluded that “studies of FACT to date provide moderate evidence to support FACT’s effectiveness toward reducing recidivism among justice-involved persons with severe mental illness.” This review also suggested that FACT is effective at promoting greater use of outpatient mental health services and at reducing hospital days. Most recently, a systematic review and meta-analysis of the FACT literature was published by Goulet et al. in 2022 and examined both forensic and health-related outcomes.^36^ The authors reported that forensic outcomes were positive and primarily driven by reductions in jail days. Positive results were also noted for utilization of outpatient mental health services, but mixed results were noted for hospitalization and health-related outcomes.

One reason why FACT reviews and studies have reported inconsistent findings is that FACT teams vary widely in their structure and operations. Simply put, it is not possible to draw definitive conclusions about the FACT model’s effectiveness in the absence of a definitive FACT model. In the absence of a standardized model, there continues to be uncertainty about FACT effectiveness and how FACT differs from ACT, if at all. For example, some authors have described FACT as a “first generation” intervention that aims to prevent criminal recidivism primarily by treating mental illness.^37^ ^-^^39^ This view suggests that FACT consists of little more than enrolling justice-involved clients into standard ACT programs, a view that was evident in New York’s implementation of forensic ACT in 2016.^40^ ^,^^41^ In contrast, other authors have described FACT as a criminologically informed hybrid that incorporates crime prevention principles into clinical team operations.^42^ ^,^^43^

To address this issue, the Substance Abuse and Mental Health Service Administration (SAMHSA) published a guideline in 2019 which listed seven “Key Components of FACT”.^44^ Three, however, pertained to ACT (e.g., high ACT fidelity, around-the-clock access, and flexible funding and implementation support). The four remaining components were (1) serving clients with histories of multiple incarcerations, (2) addressing criminogenic risks and needs, (3) having criminal justice specialists on the team, and (4) cross-system mental health and criminal justice team member training. Despite the publication of the SAMHSA guideline in 2019, subsequent reviews have suggested that FACT teams continue to vary widely in their structure and daily operations.^35^ ^,^^36^ ^,^^45^ This ongoing problem raises basic questions about how FACT differs from ACT, who FACT teams should treat, and which elements of program design and operation are necessary for FACT effectiveness.

## What are the key components of FACT?

**Figure 1.**
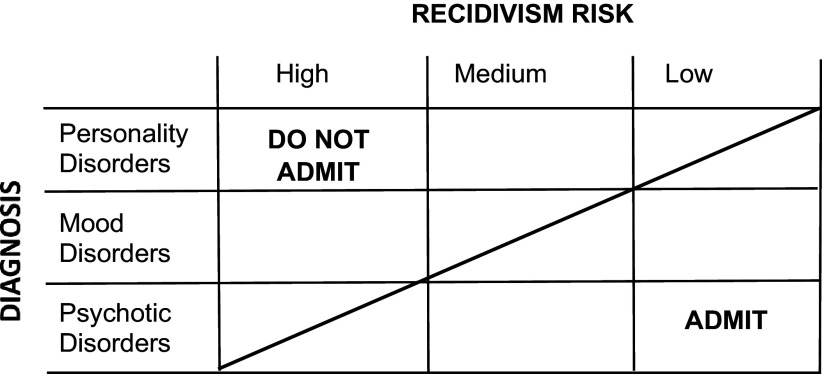
FACT enrollment framework.

To help address these questions, the authors published a 2021 review entitled “Essential Elements of Forensic Assertive Community Treatment”,^45^ based on their experience serving as FACT clinicians, researchers, and consultants. That paper presented key components of FACT which are updated and summarized as follows:

1. **A high fidelity ACT team**. Because FACT is based on the ACT model, FACT teams should meet ACT fidelity criteria with the understanding that ACT alone is generally not sufficient to prevent the arrest and incarceration of service recipients. High fidelity is recommended because high-fidelity ACT teams outperform low-fidelity ACT teams in preventing hospitalization, homelessness, and substance use, outcomes that are also important to FACT teams.^45^ In hiring FACT team members, it should be recognized that persons of color are highly over-represented within correctional settings.^46^ To help overcome cultural and language barriers to their engagement, FACT teams should make extra efforts to hire staff members whose racial/ethnic demography resembles that of their service recipients. Engagement of justice-involved clients can also be facilitated by hiring forensic peer specialists. Lastly, FACT teams should create a forensic liaison position to serve as a single point of contact for criminal justice professionals who provide legal oversight to FACT service recipients. Forensic liaisons can also play a key role in FACT teams by helping to screen referrals, and by conducting risk/need assessments as discussed below.
2. **Dual admission criteria**. Because FACT service recipients are involved in both the mental health and criminal justice systems, admission decisions must consider criteria within each system. FACT’s mental health criteria are identical to those used by ACT teams (e.g., presence of a serious mental disorder, evidence of functional impairment, frequent use of emergency room and hospital services, and lack of engagement with standard outpatient treatment). Criminal justice admission criteria, however, can vary depending on local needs. For example, they can include the history of arrest or incarceration, or current involvement in probation, parole, or a mental health court. A note of caution is warranted in considering criminal justice criteria: Some authorities assume that because FACT serves individuals with the most severe mental disorders, then it should also serve clients with the highest recidivism risk. However, individuals with the highest recidivism risk have been found to respond less well to FACT intervention compared to other FACT service recipients.^31^ ^,^^47^ To illustrate these points, Figure 1 introduces a framework that presents the relationship between psychiatric diagnosis and recidivism risk (as determined by standardized assessment tools discussed below) with respect to appropriateness for FACT enrollment. The framework presents diagnostic and risk characteristics that are most appropriate for FACT enrollment toward the lower right corner and those that are least appropriate toward the upper left corner. Enrollment decisions for individuals with diagnostic and risk characteristics near the middle of Figure 1, as represented by a dotted line, should be determined individually based on careful evaluation. For example, a high-risk client accurately diagnosed with bipolar disorder would be appropriate for admission, while a high-risk client with a questionable bipolar diagnosis may not be. Within this framework, it might be argued that prioritizing the enrollment of individuals with a low or even a moderate risk for criminal recidivism is not an appropriate use of FACT resources. The counterargument, however, is that these individuals are suffering from untreated serious mental disorders and have typically failed to engage in outpatient mental health services including ACT. Although trapped in a harmful and costly “revolving door” cycle of repeated hospitalization, homelessness, and arrest, they are more likely to benefit from FACT than individuals with high degrees of criminality.
3. **Risk/need assessment**. According to the Risk-Need-Responsivity (RNR) model, the predominant approach to crime prevention today, preventing arrest and incarceration among justice-involved individuals requires engaging them in interventions that target the risk factors driving their involvement.^48^ These risk factors, also called “criminogenic needs,” are criminal history, antisocial personality, antisocial cognition, social support for crime, family problems, work and school problems, lack of healthy leisure pursuits, and substance use. Research has consistently shown that the presence of these risk factors increases the likelihood of arrest and that addressing these risk factors effectively reduces the likelihood of arrest.^48^ ^,^^49^ Criminogenic needs can be identified through the use of standardized risk/need assessment tools such as the Level of Service/Case Management Inventory (LS/CMI),^50^ the Ohio Risk Assessment System (ORAS),^51^ or the Correctional Assessment and Intervention System (CAIS).^52^ Once they are identified, criminogenic needs must then be addressed through incorporation into FACT service recipients’ problem lists and treatment plans.
4. **Criminal justice collaboration**. Collaborating with criminal justice professionals enables shared problem-solving between mental health and criminal justice service providers, laying a foundation for the use of therapeutic alternatives to punishment.^53^ ^,^^54^ In addition, the collaboration also enables the use of legal authority to help engage individuals with untreated SMI as discussed below. It should be noted that effective collaboration requires communication as well as shared goals and values. Collaborating with criminal justice staff who value the use of punishment over problem-solving, for example, can result in increased rather than decreased arrest rates among FACT service recipients.^55^ ^,^^56^ Ideal criminal justice partners potentially include mental health court judges and specialty (i.e., mental health) probation and parole officers because they are experienced in serving people with SMI. Also, they can provide legal oversight of treatment in community settings (i.e., legal leverage).
5. **Legal leverage**. Legal leverage is the use of legal authority to promote engagement in necessary treatments and services. It is based on the practice of therapeutic jurisprudence, or the use of law as a therapeutic instrument.^57^ Examples of legal leverage include a judge offering treatment as an alternative to incarceration, or stipulating treatment as a condition of probation. Legal leverage is an essential element because FACT service recipients usually have long histories of refusing treatment, thus leaving themselves and their families to suffer the potentially catastrophic consequences of untreated SMI. Most people with SMI can be engaged by consistently providing services that are person-centered, trauma-informed, and culturally attuned. Despite clinicians’ best efforts, however, some people with SMI remain unable or unwilling to accept treatment. Many suffer from anosognosia, or unawareness of illness, and are simply not aware they are ill.^58^ To minimize perceived coercion (i.e., internal perception of being treated unfairly), it is important to utilize legal leverage in a manner described by Dr. Edward Latessa as “respectful guidance toward compliance” rather than using threats to force compliance.^49^ Offering treatment as an alternative to punishment can initiate the process of *recovery*, whereby clients may move beyond feeling forced into treatment to eventually becoming active participants in their own self-care.^59^ ^,^^60^
6. **Informed choice**. Although justice-involved adults with SMI may be offered FACT as an alternative to punishment, they must still choose whether to accept FACT versus opting to receive punitive legal consequences for their behaviors. Those who agree to FACT enrollment without having an adequate understanding of FACT services and requirements, however, may be more likely to drop out once the responsibilities of participation become clear. Extra efforts should be made to provide clear information about FACT participation and alternatives, especially given the limited literacy, motivational impairments, and cognitive limitations commonly associated with serious mental disorders.
7. **Evidence-based mental health and substance use intervention**. Legal leverage is only as effective as the treatments and services that individuals are leveraged to receive. Three deserve special mention here. First, integrated dual diagnosis treatment (IDDT) is a part of both ACT and FACT.^61^ In the authors’ experience, however, the availability and quality of chemical dependency treatment services varies widely among ACT and FACT teams alike. In addition, co-occurring substance use may be the single strongest driver of arrest and incarceration among FACT service recipients.^62^ It is therefore imperative that FACT teams provide the highest quality addiction treatment services possible. Second, in addition to having high rates of co-occurring substance use disorders, justice-involved adults with SMI typically have high rates of both treatment refusal and drug-refractory psychosis. To address these issues, FACT teams must make extra efforts to ensure that long-acting injectable medications and clozapine, respectively, are offered whenever they are clinically indicated.^63^ Ensuring optimal pharmacotherapy is particularly important for FACT teams given compelling evidence that pharmacotherapy of psychosis and mania can reduce criminal justice system involvement.^62^ Lastly, FACT teams should be prepared to offer cognitive-behavioral therapies that are designed to address antisocial cognitions and behaviors.^45^ ^,^^64^
8. **Evidence-based criminal justice intervention**. For optimal effectiveness, FACT clinicians and their collaborating criminal justice partners must each bring their respective “A games” to the table. In the field of community corrections, for example, the use of evidence-based practices is emphasized in training programs including Effective Practices in Community Supervision (EPICS),^65^ Staff Training Aimed at Reducing Arrest (STARR),^66^ and the Proactive Community Supervision model (PCS).^67^ Generally speaking, effective correctional interventions share three key features. They target criminogenic needs, they require individuals to demonstrate appropriate behaviors, and they shape behavior by extinguishing inappropriate behaviors and reinforcing appropriate ones. The principles of effective correctional intervention and associated best practices are collectively known as “what works” within the field of community corrections.^49^
9. **Shared training**. Shared training is an essential element because FACT involves collaboration between mental health and criminal justice professionals, a process that requires partnership between service providers with distinctly different values, priorities, cultures, and practices. Criminal justice staff, for example, value justice and prioritize public safety while focusing on fighting crime. Clinicians, on the other hand, value wellness and prioritize patient health while focusing on fighting illness. Shared training can take many forms including attendance at training events and cross-training, a process whereby collaborating partners teach each other about their respective service systems and practices. In addition to training, hosting informal “open house” get-togethers where FACT clinicians and their criminal justice partners can become acquainted can go a long way toward building mutual trust and cooperation.

## Current challenges for FACT model development and implementation

FACT teams continue to emerge across the United States. As of this writing, new FACT implementation initiatives are underway in several states including California, Kentucky, New York, North Carolina, and Pennsylvania. Lack of a widely accepted FACT model, however, continues to serve as a major barrier to both program implementation and outcome assessment. Steps have been taken toward FACT model standardization, most notably SAMHA’s 2019 guideline,^44^ but further efforts are needed. An important next step is to develop a valid and reliable FACT fidelity scale. The authors published an experimental FACT fidelity scale as part of their NIMH study in 2017,^31^ and they published a revised and expanded scale called the Rochester Forensic Assertive Community Treatment Scale (RFACTS) in 2021.^45^ The reliability and validity of the RFACTS, however, have yet to be tested.

Another challenge for FACT model development pertains to the question of how to define and implement “criminal justice collaboration.” Collaboration with criminal justice service providers is widely viewed as essential in serving justice-involved adults with SMI.^68^ ^,^^69^ Yet questions remain about the nature and extent of such collaboration. A key question is whether FACT teams should plan to collaborate with a single versus multiple criminal justice agencies. Collaborating with multiple agencies (i.e., *multipoint collaboration*) can ensure a steady stream of referrals, but it can also limit collaboration effectiveness due to the demands of interfacing with multiple agencies. Working in primary partnership with a single criminal justice agency such as a mental health court or a parole department (i.e., *single-point collaboration*) can potentially enable the most efficient and effective collaboration. A single agency, however, may not be able to provide an adequate number of referrals.

An additional challenge for FACT model development and implementation is to determine FACT’s target population. This is a critical question because whichever population a FACT team decides to serve will have a major impact on the team’s outcomes. FACT serves justice-involved clients, and so FACT teams can potentially receive referrals from a variety of criminal justice sources. One source that has emerged in recent years concerns individuals who have been found incompetent to stand trial (IST) and are awaiting competency restoration. Due to a lack of resources, many such individuals languish in jail for months, risking victimization and self-injurious behaviors while placing state governments at risk for civil actions. Although FACT teams can potentially provide community-based competency restoration, this approach presents the challenges of managing violence and escape risk in unstructured community settings.^70^ To minimize such risks, FACT teams that accept IST defendants should prioritize those with clear histories of SMI and without histories of repeated violent crimes.

## Recommendations for FACT research

The challenges facing FACT model development highlight the need for research in several areas. These include research to develop a reliable and valid FACT fidelity scale, to determine which individuals are most appropriate for FACT enrollment, and to identify the most critical elements of FACT design and operation. In particular, research is needed to compare the effectiveness of single-point versus multipoint collaboration designs, and to compare the impact of various forms of legal leverage versus no legal oversight on client outcomes. Lastly, given that substance use is an especially strong driver of arrest among people with serious mental disorders, research and policy-level efforts are needed to develop effective approaches to addiction treatment for FACT service recipients.
